# Dietary salt promotes ischemic brain injury and is associated with parenchymal migrasome formation

**DOI:** 10.1371/journal.pone.0209871

**Published:** 2018-12-27

**Authors:** Antje Schmidt-Pogoda, Jan-Kolja Strecker, Marie Liebmann, Christina Massoth, Carolin Beuker, Uwe Hansen, Simone König, Sarah Albrecht, Stefanie Bock, Johanna Breuer, Clemens Sommer, Nicholas Schwab, Heinz Wiendl, Luisa Klotz, Jens Minnerup

**Affiliations:** 1 Department of Neurology with Institute of Translational Neurology, University of Münster, Münster, Germany; 2 Department of Anesthesiology and Intensive Care Medicine, University of Münster, Münster, Germany; 3 Institute of Musculoskeletal Medicine, University of Münster, Münster, Germany; 4 IZKF Core Unit Proteomics, University of Münster, Münster, Germany; 5 Department of Neuropathology, University of Mainz, Mainz, Germany; University Hospital-Eppendorf, GERMANY

## Abstract

Sodium chloride promotes vascular fibrosis, arterial hypertension, pro-inflammatory immune cell polarization and endothelial dysfunction, all of which might influence outcomes following stroke. But despite enormous translational relevance, the functional importance of sodium chloride in the pathophysiology of acute ischemic stroke is still unclear. In the current study, we show that high-salt diet leads to significantly worse functional outcomes, increased infarct volumes, and a loss of astrocytes and cortical neurons in acute ischemic stroke. While analyzing the underlying pathologic processes, we identified the migrasome as a novel, sodium chloride-driven pathomechanism in acute ischemic stroke. The migrasome was previously described *in vitro* as a migrating organelle, which incorporates and dispatches cytosol of surrounding cells and plays a role in intercellular signaling, whereas a pathophysiological meaning has not been elaborated. We here confirm previously reported characteristics of the migrasome *in vivo*. Immunohistochemistry, electron microscopy and proteomic analyses further demonstrate that the migrasome incorporates and dispatches cytosol of surrounding neurons following stroke. The clinical relevance of these findings is emphasized by neuropathological examinations, which detected migrasome formation in infarcted brain parenchyma of human stroke patients. In summary, we demonstrate that high-salt diet aggravates stroke outcomes, and we characterize the migrasome as a novel mechanism in acute stroke pathophysiology.

## Introduction

High dietary salt plays a significant role in the pathogenesis of arterial hypertension and also promotes the development of vascular, myocardial and renal fibroses through blood pressure-independent mechanisms [1]. In contrast to these long-term cardiovascular effects, even a short-term increase in dietary salt is sufficient to boost autoimmune diseases [2–5]. In a mouse model of multiple sclerosis, for instance, transient high-salt diet resulted in significantly worse outcomes through induction of Interleukin-17-producing CD4-positive T helper cells (T_H_17 cells) [4,5]. Our own group found a strong pro-inflammatory macrophage phenotype with augmented pro-inflammatory cytokine production, upregulation of immune-stimulatory molecules and antigen-independent T cell proliferation in response to sodium chloride-delivery during experimental autoimmune encephalomyelitis [3]. It is altogether well-established that even a short-term increase in salt intake triggers profound alterations in peripheral immune cell polarization [3–5].

Apart from promoting autoimmunity, high dietary salt causes endothelial dysfunction of peripheral and cerebral arteries by suppression of the endothelial nitric oxide (NO) production through inhibitory phosphorylation of endothelial NO synthase (eNOS) [6,7]. A recent study moreover demonstrated that mice on high-salt diet developed significant cerebral hypoperfusion and disturbed cerebral microcirculation due to endothelial dysfunction [8]. Cerebral hypoperfusion is a primary cause of ischemic stroke. But independent of the stroke etiology, hypoperfusion and disturbed microcirculation critically reduce the oxygen and glucose supply of potentially salvageable brain tissue, leading to electric failure of membrane-bound ion channels, acidosis, accumulation of reactive oxygen species and activation of proteolytic enzymes, thus aggravating ischemic injury [9,10].

Cerebral ischemia triggers an immediate local immune response with secretion of pro-inflammatory cytokines and upregulation of chemokines like monocyte-chemoattractant protein 1 (MCP-1), which attract further resident and immigrating immune cells to the ischemic area [11]. These leukocytes, which migrate into ischemic brain parenchyma within the first hours after stroke, induce neuronal cell death and augment ischemic injury [12,13]. Considering the harmful effects of immigrating leukocytes following stroke, a sodium chloride-induced amplification of the immune response might further aggravate stroke outcomes. Apart from that, it is conceivable that salt exacerbates stroke outcomes through endothelial dysfunction, cerebral hypoperfusion and disturbed microcirculation.

With respect to the huge disease burden of stroke, increasing salt intake of the population [14], and sodium chloride-containing fluid substitution in stroke patients, the functional role of sodium chloride in stroke pathophysiology has got an enormous translational relevance. Nonetheless, the functional importance of sodium chloride in the pathophysiology of acute ischemic stroke is still unclear. In the current study, we investigated the effects of high-salt diet in a mouse model of ischemic stroke. Our results show significantly worse functional outcomes, increased infarct volumes, and a loss of astrocytes and cortical neurons following high-salt diet. But most importantly, we firstly describe the migrasome, which incorporates and dispatches the cytosol of surrounding neurons, as a novel, sodium chloride-driven mechanism in stroke pathophysiology.

## Results

### Sodium chloride aggravates functional and structural outcomes after ischemic stroke

**Fig 1:**
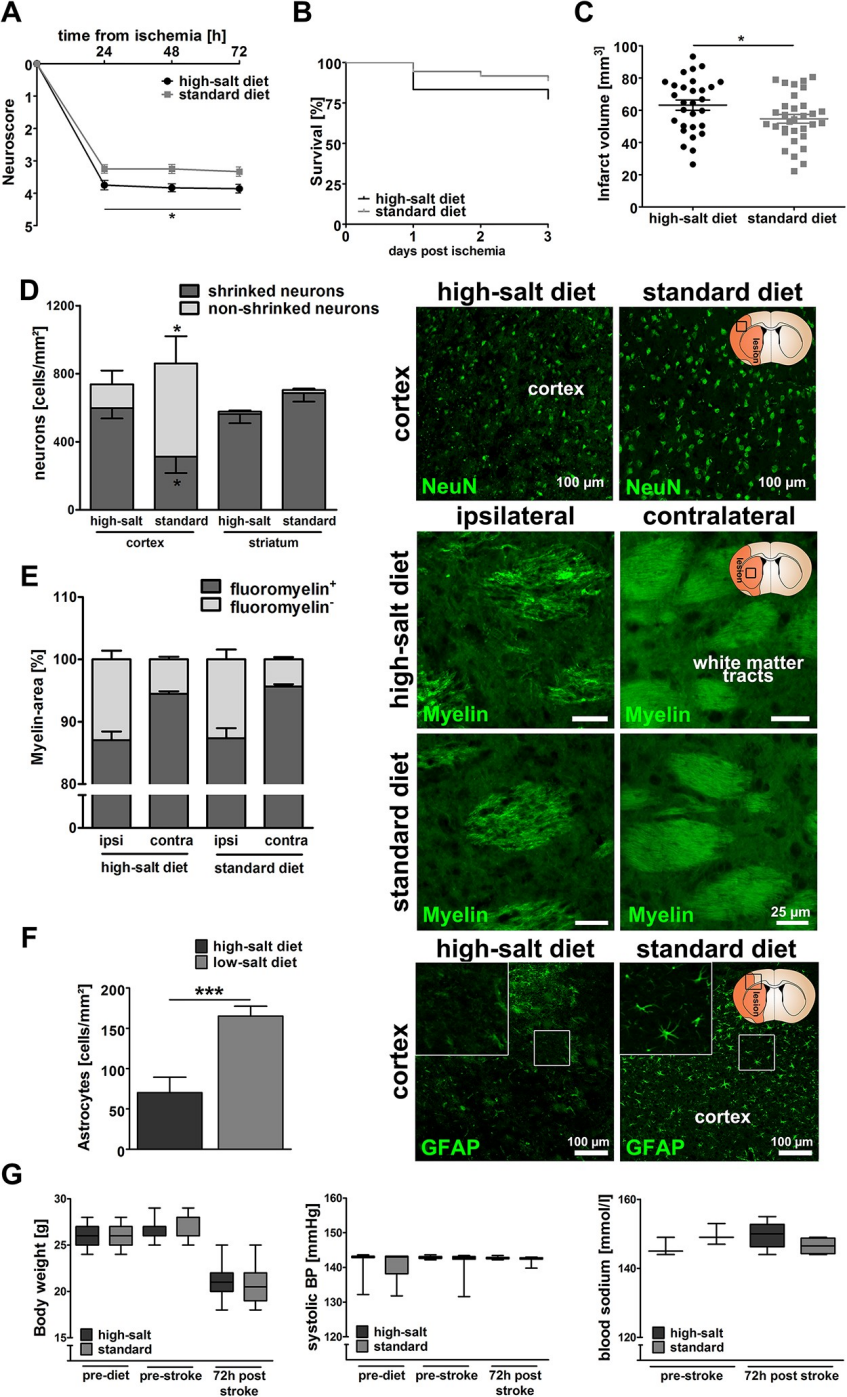
Sodium chloride aggravates functional and structural outcomes after ischemic stroke. **(A)** Neurological deficit score assessment showed significantly worse functional outcomes following high-salt diet (p<0.05, 2way ANOVA, n = 36 per group). **(B)** Mortality rates did not significantly differ between both groups (p = 0.19, Log-rank (Mantel-Cox) Test, n = 36 per group). **(C)** Infarct volumes were significantly increased after high-salt diet (64.2 mm^2^ ± 3.1 mm^2^ vs. 54.7 mm^2^ ± 2.7 mm^2^, p<0.05, t-test, n = 28 to 31 per group). **(D)** Immunohistochemical analyses revealed a significant increase in the number of shrinked cortical neurons following high-salt diet, and vice versa a reduced number of intact cortical neurons (p<0.05, t-test, n = 8 to 11 per group). NeuN-staining was used to visualize intact and shrinked neurons. **(E)** The extent of fluoromyelin-positive myelin areas was similar in both groups (p = 0.88, t-test, n = 20 to 22 per group) Fluoromyelin-staining was employed to visualize white matter tracts in the ispilateral and contralateral hemisphere of both groups. **(F)** High-salt diet induced a significant loss of GFAP^+^-astrocytes in the periinfarct region (p<0.001, t-test, n = 10 to 11 per group). **(G)** Body weight and systolic blood pressure were not significantly influenced (p = 0.95 and p = 0.83 2way ANOVA, n = 36 per group). Blood sodium levels did not significantly differ between both groups prior to MCAO and three days after MCAO (p = 0.19 and p = 0.18, t-test, n = 3 to 6 per group).

Seventy-two adult male C57BL/6 mice received either high-salt diet containing 4% NaCl (ssniff, Germany) and tap water containing 1% NaCl ad libitum (high-salt, n = 36) or standard diet and tap water ad libitum (standard, n = 36), as previously described [3,4]. Seven days after the initiation of high-salt diet, ischemic stroke was induced by transient middle cerebral artery occlusion (MCAO) as previously described [15,16]. To evaluate whether high-salt diet had an impact on functional outcomes, neurological deficit score assessment was performed according to a modification of Menzies neuroscore [15], which showed significantly worse outcomes following high-salt diet (p<0.05, 2way ANOVA, Fig 1A). Mortality rates did not significantly differ between both groups (p = 0.19, Log-rank (Mantel-Cox) Test, Fig 1B). We next aimed to identify the structural correlates of worse functional outcomes among animals receiving high-salt diet. Infarct volume assessment demonstrated significantly increased infarct volumes as structural correlates of worse functional outcomes after high-salt diet (64.2 mm^2^ ± 3.1 mm^2^ vs. 54.7 mm^2^ ± 2.7 mm^2^, p<0.05, t-test, Fig 1C). Immunohistochemical analyses revealed a significant increase of shrunk and a decrease of intact cortical neurons following high-salt diet (p<0.05, t-test, Fig 1D), whereas the extent of fluoromyelin-positive myelin areas did not differ (p = 0.88, t-test, Fig 1E), thus indicating that high dietary salt causes worse outcomes through neuronal rather than white matter damage. As a previous study had shown a massive loss of astrocytes after excessive sodium delivery in hyponatremia [17], we quantified astrocytes in the periinfarct region. In accordance with those previous findings, our results showed that high-salt diet induced a significant loss of astrocytes in the periinfarct region (p<0.001, t-test, Fig 1F).

Considering that systemic effects of sodium chloride might have contributed to worse functional outcomes, we monitored physiological parameters. The body weight did not differ between both experimental groups (p = 0.95, 2way ANOVA, Fig 1G). To exclude sodium-induced hypertension as mediating factor, we monitored the blood pressure, which did not change in response to high-salt diet (p = 0.83, 2way ANOVA, Fig 1G). As previously described [8], blood sodium levels did also not differ between mice on high-salt diet and mice on standard diet (p = 0.19 and p = 0.18 prior to MCAO and three days after MCAO, t-test, Fig 1G), thus excluding severe hypernatremia as an underlying cause of worse outcomes.

These findings demonstrate that sodium chloride promotes damage of cortical neurons and worsens neurological outcomes after stroke.

### Sodium chloride induces vesicle formation of microglia/macrophages

**Fig 2:**
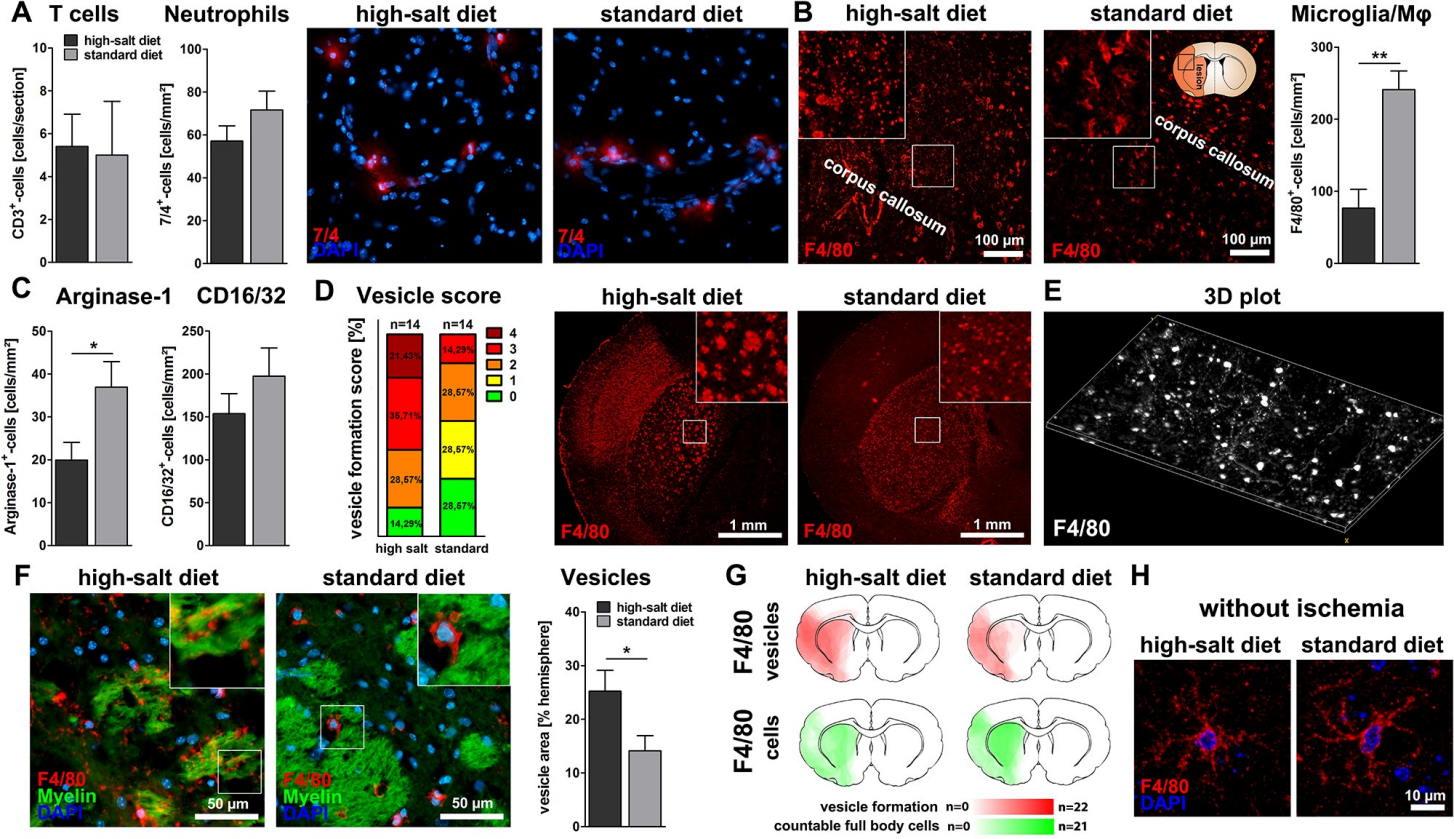
Sodium chloride induces vesicle formation of microglia/macrophages. **(A)** The total number of infiltrating 7/4^+^-neutrophils and CD3^+^-T cells was not affected (p = 0.16 and p = 0.61, t-test, n = 15 per group (7/4) and n = 5 per group (CD3)). **(B)** The number of F4/80^+^-microglia/macrophages was significantly reduced after high-salt diet (p<0.01, t-test, n = 15 per group). **(C)** Arginase-1-stainings illustrated a significant decrease of Arginase-1^+^-anti-inflammatory cells following high-salt diet (p<0.05, t-test, n = 15 per group). High-salt diet did not influence the number of CD16/32^+^-pro-inflammatory cells (p = 0.28, t-test, n = 16 per group). **(D)** Quantification of vesicle formation among F4/80^+^-cells according to a previously defined score revealed increased vesicle formation following high-salt diet (score 0: No vesicles, 1: few vesicles, 2: occasional vesicle formation, 3: vesicle formation covering at least half of the striatum, 4 extreme vesicle formation, n = 14 per group). **(E)** Three-dimensional plot illustrating the distribution of vesicles and cytoplasmic expansions within the primary motor cortex. **(F)** The total area occupied by vesicles was significantly larger after high-salt diet (p<0.05, t-test, n = 16 per group). **(G)** Heat maps illustrate the distribution of F4/80^+^-vesicles (red) and intact F4/80^+^-microglia/macrophages (green) in both experimental groups. **(H)** In sham operated animals, high-salt diet did not induce microglial vesicle formation.

Since sodium chloride was reported to influence the occurrence and phenotype of immune cells in autoimmune diseases [3–5], we analyzed the impact of high-salt diet on immigrating and resident immune cells after cerebral ischemia. High-salt diet did not alter the number of immigrating neutrophils and T cells after cerebral ischemia (p = 0.16 and p = 0.61, t-test, Fig 2A). As the total number of immigrating T cells after stroke is known to be relatively small [18], and a previous study demonstrated an expansion of T_H_17 cells in the distal small intestine and lymphoid organs, but not in brain or meninges after high-salt diet [8], we did not further quantify T cell subsets in brain parenchyma. We proceeded with a characterization of microglia/macrophages. The total number of F4/80^+^-microglia/macrophages was significantly reduced after high-salt diet (p<0.01, t-test, Fig 2B). In particular, the number of cells expressing Arginase-1, an exemplary marker for anti-inflammatory microglia/macrophages [16,19], was reduced following high-salt diet (p<0.05, t-test, Fig 2C). CD16/32^+^-cells, indicating pro-inflammatory microglia/macrophages [19,20], were unaltered (p = 0.28, t-test, Fig 2C). Completely unexpected, we spotted abundant F4/80^+^-vesicles with long tubular cytoplasmic expansions in the ischemic hemispheres of animals that received high-salt diet. We pursued this serendipitous observation and compared the extent of vesicle formation between both experimental groups. Vesicle formation was massively increased following high-salt diet (p<0.05, t-test, Fig 2D–2G), but could also be detected after standard diet. In sham animals on high-salt diet, we did not detect any microgial vesicles (Fig 2H).

In summary, these findings show that high-salt diet leads to a reduced number of microglia/macrophages, and, most interestingly, induces a not yet described massive vesicle formation of microglia/macrophages following stroke.

### Characterization of F4/80^+^-vesicles as migrasomes dispatching neuronal cytoplasm

**Fig 3:**
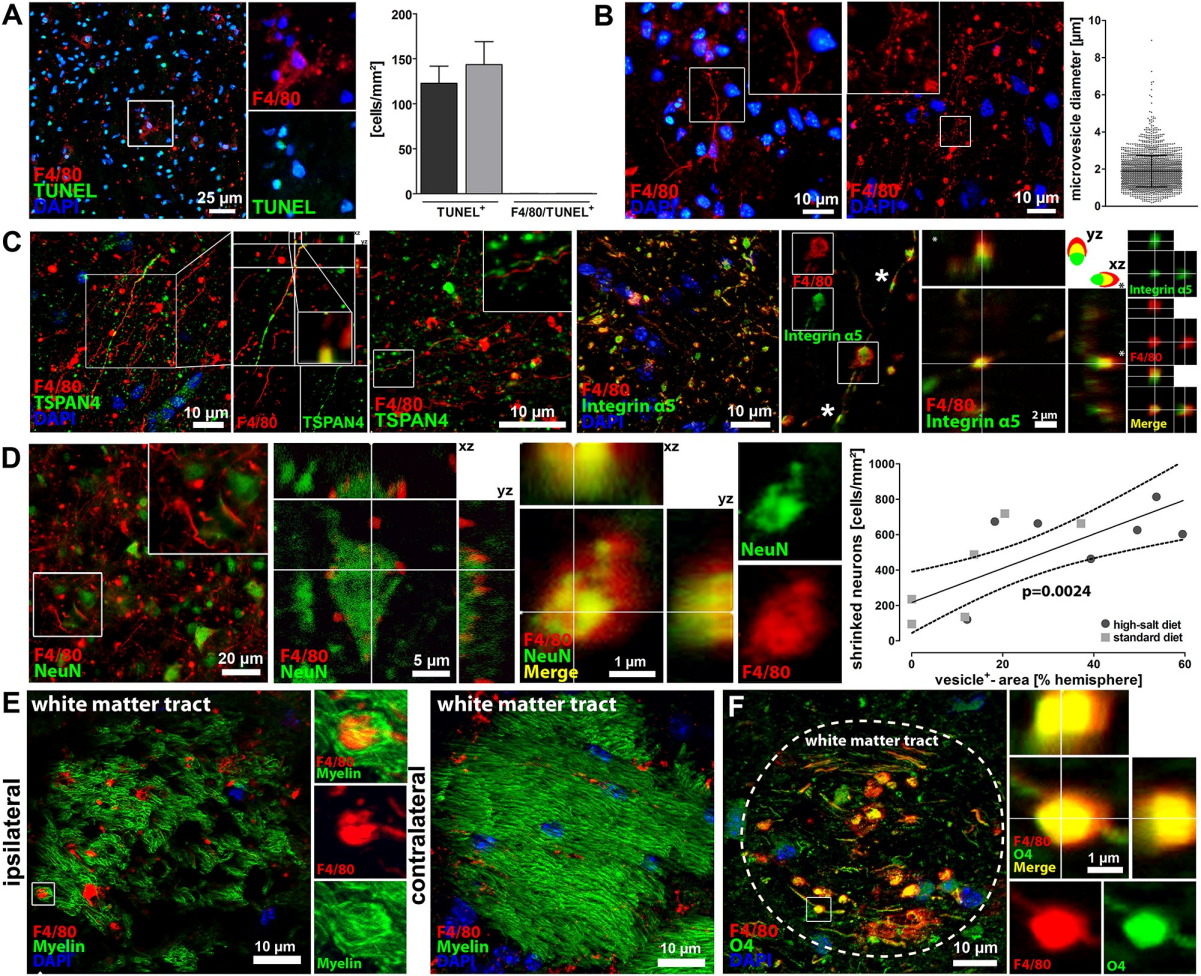
Characterization of F4/80^+^-vesicles as migrasomes dispatching neuronal cytoplasm. **(A)** The previously identified F4/80^+^-vesicles did not interact with TUNEL, thus rebutting the hypothesis that these vesicles were merely apoptotic blebs. **(B)** We measured the diameter of > 2300 vesicles and calculated a mean diameter of 1,87 ± 0,18 μm, which is in the range of the migrasome. **(C)** Immunohistochemistry and confocal microscopy demonstrated that the F4/80^+-^vesicles expressed the characteristic migrasome-markers TSPAN4 and integrin α5, which provides evidence that the identified vesicles belong to the migrasome. Furthermore, our findings confirm the previously *in vitro* described polarized expression pattern of TSPAN4 and the migrasomal enriched expression of integrin a5 with occasional small integrin-positive puncta (asterisks), which have been shown to be sites of migrasome formation *in vitro*. **(D)** Intact and shrinked NeuN^+^-neurons were found in close vicinity to the migrasome, and confocal microscopy visualized neuronal fragments within the migrasome, thus suggesting that the migrasome incorporates the cytosol of surrounding neurons. Correlation analyses moreover revealed a positive correlation between vesicle occurrence and neuronal loss (p<0.01, linear regression, n = 7 per group). **(E)** Immunohistochemistry and confocal microscopy demonstrated that the migrasome infiltrates Fluoromyelin^+^-white matter tracts. **(F)** Immunohistochemistry and confocal microscopy also demonstrated that the migrasome interacts with O4^+^-oligodendrocytes.

Based on the surprising finding of massive vesicle formation after high-salt diet, we next aimed to characterize the observed vesicles. We firstly hypothesized that the vesicles might be apoptotic blebs, but the lack of TUNEL-co-localization (Fig 3A) did not support this hypothesis. The mean vesicle diameter was 1,87 ± 0,18 μm (Fig 3B), which exceeds the dimension of exosomes and microvesicles, but is in the range of migrasomes [21,22]. Migrasomes were recently identified as novel organelles, which incorporate cytosolic material of surrounding cells and express Tetraspanin-4 (TSPAN4) and integrin α5 as characteristic markers [22,23]. Indeed, we found that the high-salt diet-induced F4/80^+^-vesicles after cerebral ischemia expressed TSPAN4 and integrin α5 (Fig 3C). We also found the previously described polarized expression pattern of TSPAN4 and integrin α5, with predominant TSPAN4-expression on the upper side and prevailing integrin α5-expression on the bottom side of the migrasome (Fig 3C) [23]. The vesicles also exhibited long, tubular cytoplasmic expansions that were described as characteristic morphological features of migrasomes called retraction fibers (Fig 3C) [22,23].

Since high-salt diet augmented both, migrasome formation and shrinkage of neurons, we hypothesized that migrasome formation and neuronal damage might be associated. To verify this hypothesis, we performed immunohistochemical co-stainings of F4/80 and Neuronal Nuclei (NeuN), which is expressed in nuclei and cytoplasm of neurons, and found an impressive co-localization of F4/80^+^-migrasomes and NeuN-fragments (Fig 3D). In addition, we found a significant correlation between the number of shrunk neurons and the extent of migrasome formation (p<0.01, linear regression, n = 7 per group, Fig 3D). These findings suggest that cytoplasmic material of surrounding neurons is translocated into the migrasome. Such incorporation of cytosol of surrounding cells by migrasomes was previously reported [22,24].

Our next goal was to investigate, if the migrasome also interacts with white matter. Immunohistochemical stainings showed in fact a co-localization of F4/80^+^-migrasomes and Fluoromyelin (Fig 3E) and oligodendrocyte-protein O4 (Fig 3F), suggesting a potential interaction between the migrasome and white matter. However, as reported above, quantification of myelin areas did not show an impact of high-salt diet on white matter damage following cerebral ischemia (differ (p = 0.88, t-test, Fig 1E).

**Fig 4:**
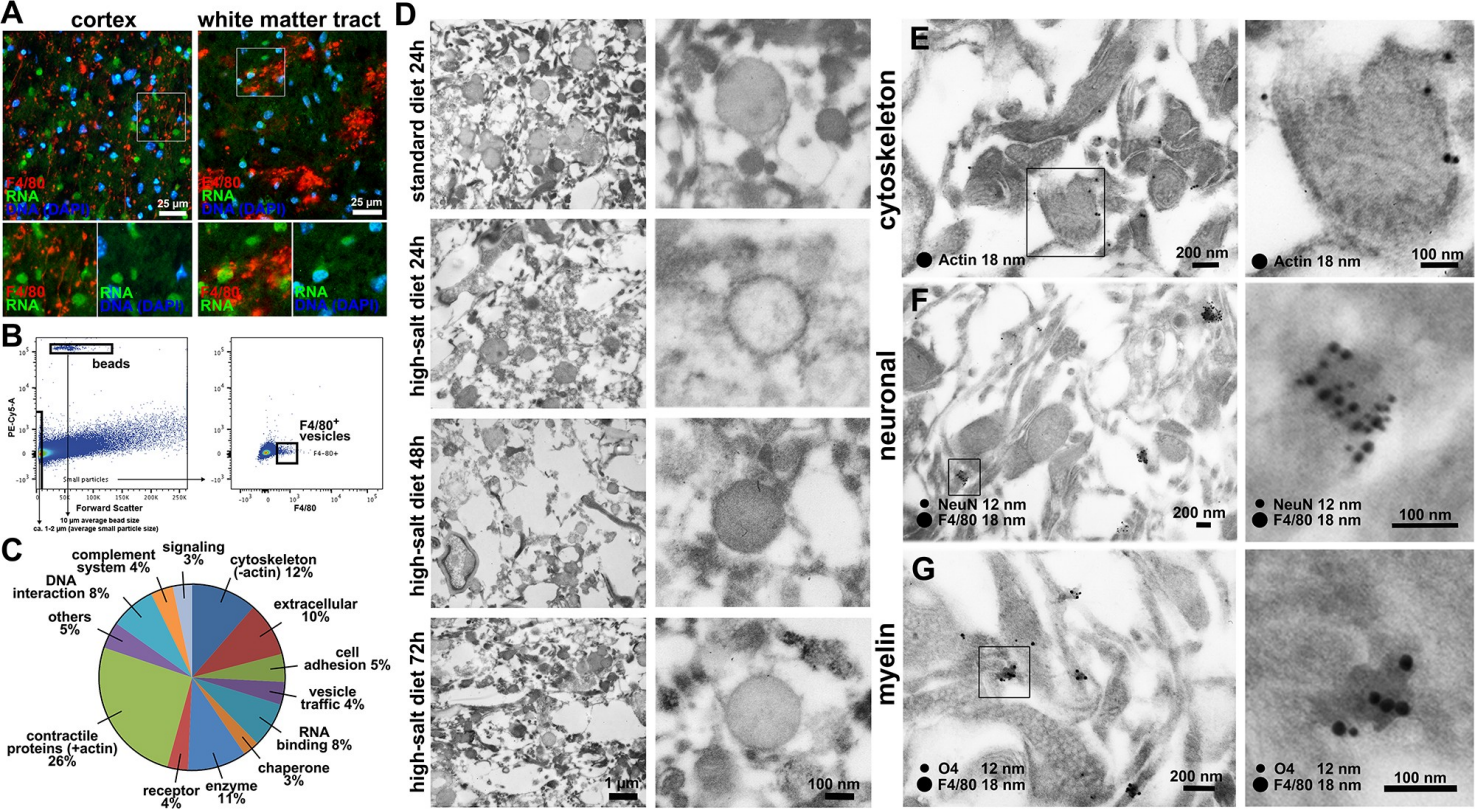
Identification of migrasome components. **(A)** Co-staining of F4/80 and RNA did not show RNA-signaling within the migrasome either in cerebral cortex or white matter tracts. **(B)** Gating strategy: All particles smaller than 2 μm in size compared to the added beads were sorted for F4/80^+^-signal. Quantification was performed by comparing the number of beads with the number of F4/80-positive particles. Quantification revealed a higher amount of F4/80^+^-vesicles in high-salt diet group when compared to the standard diet. **(C)** Proteomic analyses demonstrated that the migrasome was mainly composed of contractile proteins actin and myosin, cytoskeleton and annexin proteins, which were previously described to be involved in trafficking and organization of vesicles [25]. **(D)** Electron microscopy confirmed the typical morphological characteristics of the migrasome on ultrastructural level. Time course analyses revealed an increasing degree of extracellular tissue damage within the first 72 h after stroke. Vesicle close-ups reveal cup-like structures carrying spherical structures in all investigated time-points and treatments. **(E)** Immunogold-staining shows actin-postive signals especially in the outer layer of the vesicle-like structures. **(F)** Co-staining of F4/80 and NeuN confirms fluorescence microscopic observations and shows a distinct clustering of F4/80 and NeuN-signals in small vesicle-like structures. **(G)** F4/80 and O4-staining also reveals a solid co-localization in node-like structures.

We next aimed to identify further components of the migrasome. RNA-stainings did not show RNA-signals within the migrasome (Fig 4A), which rules out an involvement in mRNA trafficking. For a more detailed analysis of migrasome components, we isolated the migrasome from infarcted brain parenchyma using fluorescence-activated cell sorting (FACS) and performed proteomic analyses. In accordance with previous descriptions of the migrasome as a migrating organelle [22,24], proteomic analyses demonstrated that the migrasome is mainly composed of contractile proteins actin and myosin, cytoskeleton and annexin proteins (Fig 4B).

To reinforce these observations on an ultrastructural level, we employed electron microscopy, which confirmed the typical morphological characteristics of the migrasome (Fig 4C and 4D). Time course analyses moreover illustrated an increasing degree of extracellular tissue damage within the first 72 h after stroke (Fig 4C and 4D). As fluorescence microscopy and proteomics revealed contractile proteins as well as neuronal and myelin signals within F4/80^+^-vesicles, we performed electron microscopic immunohistochemical labeling of these proteins. Indeed, immunogold-labeling showed actin-positive stainings especially within the outer layers of the vesicles (Fig 4E). F4/80-co-staining with NeuN and myelin-marker O4 showed concentrated clustering of F4/80 with NeuN (Fig 4F) and F4/80 with O4 (Fig 4G).

In summary, these findings confirm that high-salt diet triggers migrasome formation in cerebral ischemia.

### Sodium chloride induces microglial migrasome formation and promotes a pro-inflammatory polarization of microglia, whereas the cells’ metabolism remains unaltered

**Fig 5:**
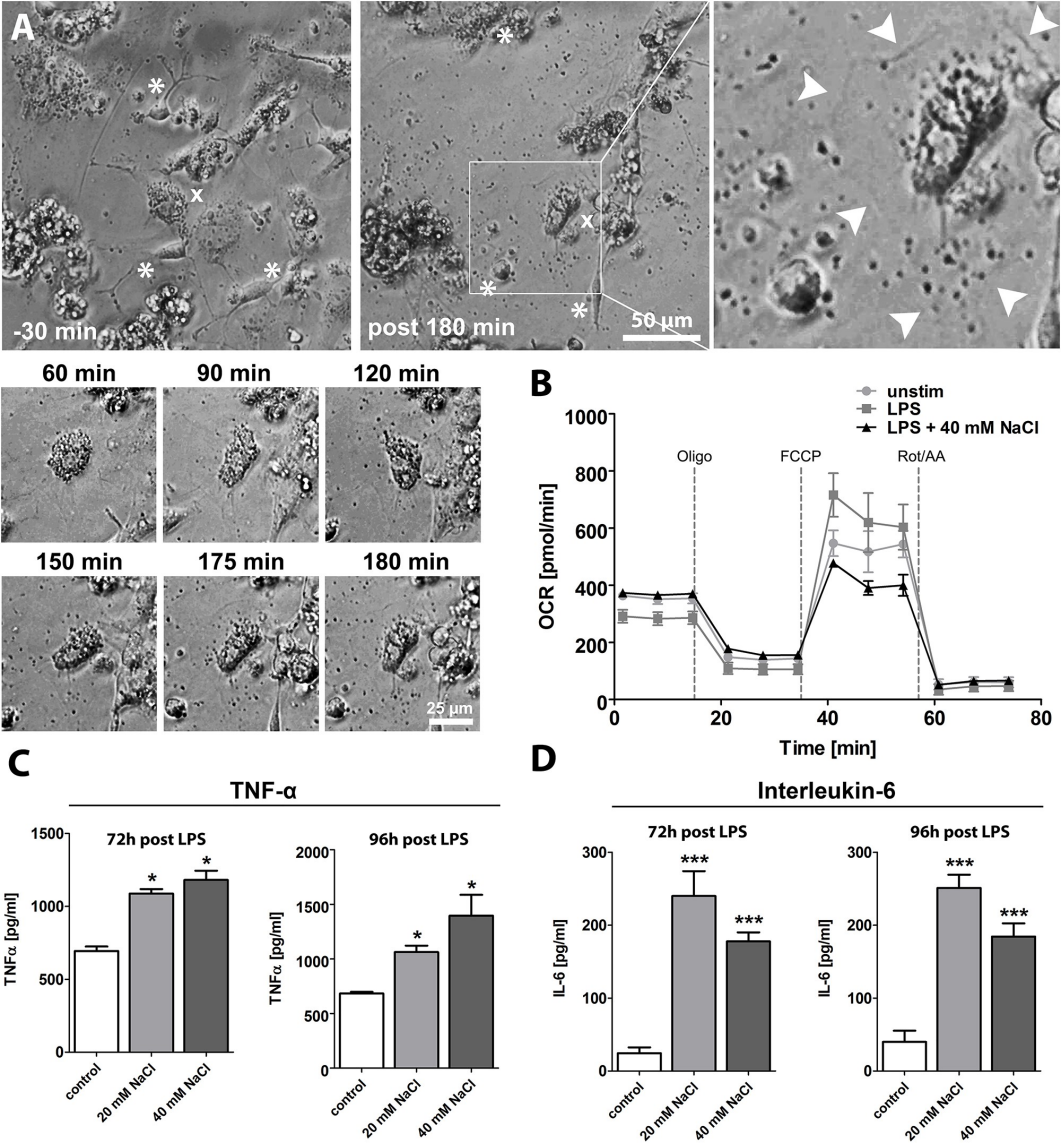
Sodium chloride induces microglial migrasome formation and promotes a pro-inflammatory polarization of microglia, whereas the cells’ metabolism remains unaltered. **(A)** After application of 20 mM NaCl to a primary murine microglia and astrocyte co-culture, we firstly observed the appearance of vesicle-like structures and subsequently, we observed morphological changes of microglia and astrocytes. Note that images have been enhanced regarding contrast to make structural differences better visible. **(B)** Sodium chloride delivery did not influence the mitochondrial respiration of microglia (p = 0.31, 2way ANOVA). **(C)** Incubation of a murine microglia cell culture with lipopolysaccharide (LPS) plus either solvent or sodium chloride (20 mM and 40 mM) for 72 h to 96 h resulted in a significantly increased concentration of pro-inflammatory cytokine TNF-α in the supernatant (p<0.05, 1way ANOVA). **(D)** Incubation of a murine microglia cell culture with lipopolysaccharide (LPS) plus either solvent or sodium chloride (20 mM and 40 mM) for 72 h to 96 h resulted in a significantly increased concentration of pro-inflammatory cytokine IL-6 in the supernatant (p<0.001, 1way ANOVA).

Based on our previous observations that high-salt diet induces massive migrasome formation of microglia/macrophages and leads to a reduced number of microglia/macrophages and astrocytes after cerebral ischemia, we further aimed to investigate the effect of sodium chloride on microglia and astrocytes *in vitro*. We first incubated a murine microglia and astrocyte co-culture with 20 mM sodium chloride and employed time-lapse microscopy over the course of 3.5 hours (Fig 5A and S1 Video). In accordance with our immunohistochemical *in vivo*-findings, we observed the appearance of migrating vesicle-like structures in response to sodium chloride application (Fig 5A and S1 Video). The mean diameter of these vesicle-like structures observed *in vitro* was 1,9 ± 0,4 μm, which is in the range of the migrasome. Therefore, we hypothesize that sodium chloride is able to induce migrasome formation independent of a further distinct pathophysiological stimulus.

Based on the finding that sodium chloride prompts microglia to generate migrasomes, we next aimed to investigate, if sodium chloride also influences cell functions such as the immune metabolism and cytokine production of microglia. For this purpose, we incubated a murine microglia cell culture with lipopolysaccharide (LPS), which was added to simulate a pro-inflammatory environment similar to the postischemic inflammation after stroke, plus either solvent or sodium chloride (40 mM) for 72 h and performed a functional analysis of the mitochondrial respiratory capacity. These metabolic assays included measurements of the cells’ basal respiration, measurements of the maximal respiration in response to FCCP after Oligomycin-mediated suppression and a determination of the spare respiratory capacity, i.e. the difference between maximal respiration and basal respiration. As illustrated by Fig 5B, sodium chloride delivery did not influence the metabolism of microglia (p = 0.31, 2way ANOVA, Fig 5B).

Since previous studies have demonstrated that sodium chloride promotes a pro-inflammatory polarization of macrophages and T cells [3–5], we hypothesized that sodium chloride might induce a pro-inflammatory phenotype of microglial cells. To verify this hypothesis, we incubated murine primary microglial cells with lipopolysaccharide (LPS) plus either solvent or sodium chloride (20 mM and 40 mM) for 72 h to 96 h, and performed enzyme-linked immunosorbent assays (ELISA) for TNF-α and IL-6 cytokine detection. As demonstrated by Fig 5C, the concentration of pro-inflammatory cytokine TNF-α in the supernatant was significantly increased 72 h and 96 h after sodium chloride delivery (p<0.05, 1way ANOVA, Fig 5C). Similarly, the concentration of pro-inflammatory cytokine IL-6 was significantly increased 72 h and 96 h after sodium chloride delivery (p<0.001, 1way ANOVA, Fig 5D), thus supporting the hypothesis that sodium chloride promotes a pro-inflammatory polarization of microglial cells, which may have contributed to worse functional outcomes following MCAO in our *in vivo*-experiments.

In summary, our experiments firstly demonstrate that sodium chloride induces a pro-inflammatory polarization of microglial cells, which represent the most numerous immune cell population after ischemic stroke [18].

### Migrasome formation in human stroke specimens

**Fig 6:**
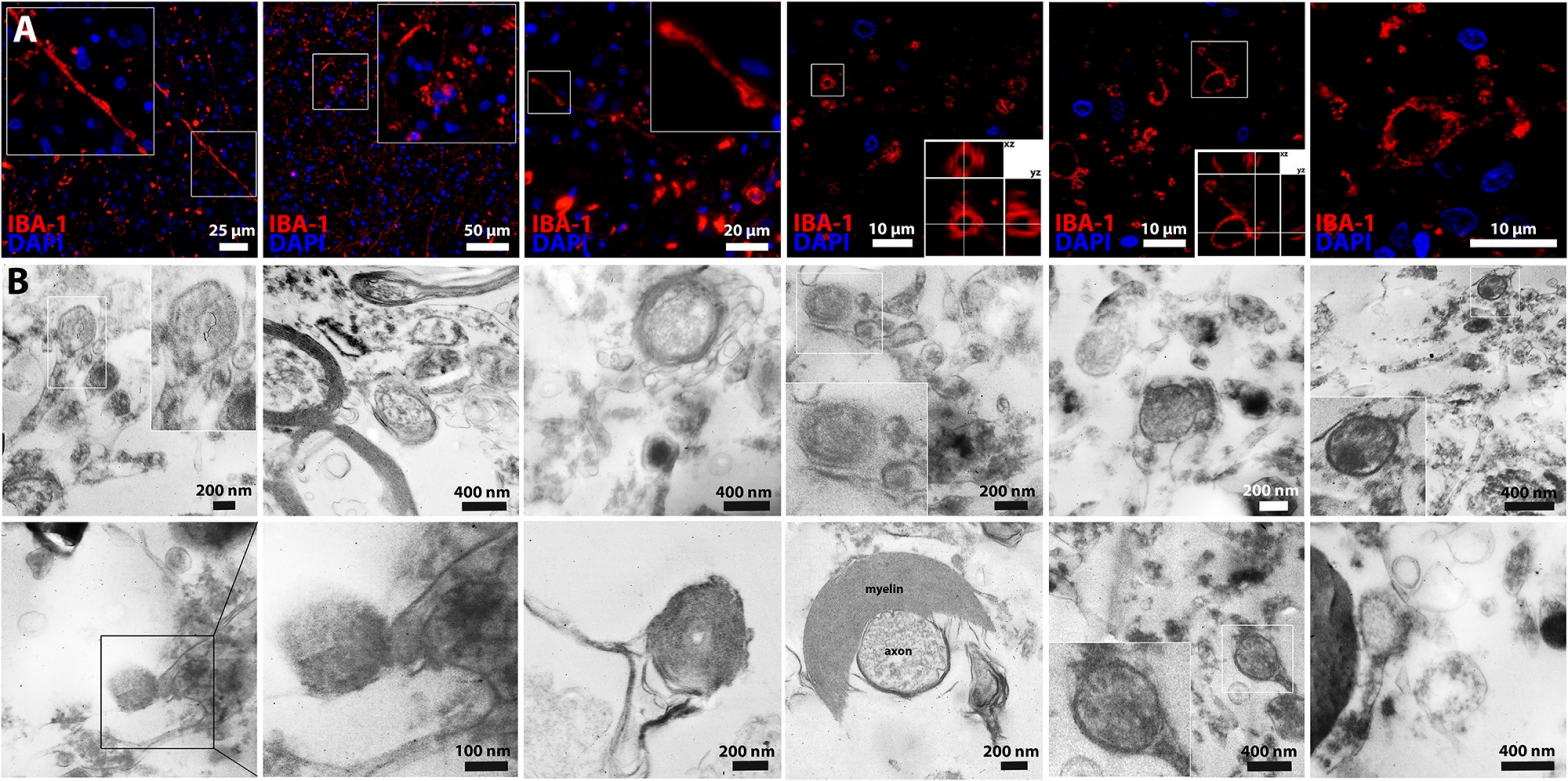
Migrasome formation in human stroke specimens. **(A)** Iba1 (microglia/macrophage)-stainings showed cells with unequivocal morphological features of migrasomes in postmortem brain tissue of patients who died within a few days after having a stroke (< 7 days). **(B)** Electron microscopy of human stroke specimen also showed similar characteristics of the migrasome on the ultrastructural level within infarcted tissue.

We next aimed to evaluate the relevance of migrasomes for humans. For this purpose, we examined postmortem brain tissue of five patients who died within a few days after having a stroke (< 7 days). In ischemic tissue, Iba1 (microglia/macrophage)-stainings showed cells with unequivocal morphological features of migrasomes (Fig 6A), whereas no such structures could be detected in normal brain tissue. We stained postmortem tissue of five stroke patients and re-proved the existence of the migrasome in the autoptic tissue of four out of five patients. Furthermore, electron microscopy revealed similar morphological patterns including cup-like embedding of vesicles, retraction fibers or myelin-interaction of the migrasome within the infarcted area (Fig 6B). A limitation of our analysis is that the premortal salt intake of the patients, whose autoptic tissue we examined, could not be traced back. However, with respect to common dietary patterns of stroke patients, we assume that the salt intake of those patients was rather high. Apart from that, our animal experiments demonstrated that migrasomes were also detected in mice that received a standard diet, whereas high-salt diet increased the extent of migrasome formation.

Altogether, our neuropathological examinations demonstrate that migrasomes are involved in the pathogenesis of brain injury after cerebral ischemia in humans, thus highlighting the clinical relevance of our study.

## Discussion

In this study, we show that high-salt diet promotes ischemic injury of the central nervous system and induces massive migrasome formation in ischemic brain parenchyma. Migrasomes were previously described *in vitro* as bulb-like structures that migrate along tubular cytoplasmic expansions, called retraction fibers, and incorporate and dispatch cytosol of surrounding cells [22,24]. The function of migrasomes has not yet been conclusively elaborated, but since other cells were observed to follow the pathways of migrasomes and finally internalize the migrasomes, it was assumed that migrasomes play a role in cell-cell communication [22,24]. In accordance with these previous reports of the migrasome, we found bulb-like structures, partly attached to long cytoplasmic expansions and Tetraspanin4- and Integrin α5-expression as characteristic features of the migrasome. The proteomic evidence of actin as a major component of the migrasome in our study is also in line with previous findings demonstrating that actin polymerization is a prerequisite for migrasome formation [22]. Proteomic detection of actin and myosin as part of the migrasome moreover supports the thesis of an active incorporation and transport of cytosolic material. The occurrence of shrunk neurons as well as intact neurons in close vicinity to the migrasome, the evidence of neuronal fragments within the migrasome, and the correlation between migrasome formation and neuronal loss suggest that the migrasome incorporates and dispatches cytosol of surrounding neurons. Two different scenarios are possible: On the one hand, migrasomes might incorporate the cytosol of intact neurons, thereby inducing neuronal death and aggravating ischemic cell damage. On the other hand, migrasomes might carry off fragments of damaged neurons, thus fulfilling a “cleavage function”. In the latter scenario, migrasome formation would be a consequence rather than a mediating factor of salt-induced neuronal damage. Similarly, increased migrasome formation in response to sodium chloride delivery in our *in vitro*-experiments might be attributed to sodium chloride-induced cell damage, which in turn promoted migrasome formation.

As demonstrated by our *in vitro*-experiments, high-salt diet promoted a pro-inflammatory microglia polarization. This observation is in line with previous findings in other disease models, which demonstrated a meaningful influence of sodium chloride on the phenotype of hematogenous immune cells [3–5]. In a model of experimental autoimmune encephalitis (EAE), for instance, sodium chloride was reported to aggravate CNS autoimmunity by pro-inflammatory macrophage polarization [3]. Other studies focused on T cells and worked out that sodium chloride drives autoimmune diseases by an induction of interleukin (IL)-17-producing CD4^+^ helper T cells (T_H_17 cells) [4,5]. In a recently published study, excess dietary salt was moreover reported to cause endothelial dysfunction and cognitive impairment through a gut-initiated T_H_17 response [8]. The number of T_H_17 cells in brain and meninges was not affected, though [8]. Following stroke, the number of immigrating T cells is generally small [18] and in our study, T cell immigration was not influenced by high-salt diet. Instead, we report for the first time that sodium chloride influences the phenotype of microglial cells, which represent the most numerous immune cell population following ischemic stroke [18]. The observation that salt induces a pro-inflammatory microglia polarization is of particular translational interest, since the postischemic inflammation is one of the most frequently addressed therapeutic targets following experimental stroke, and immunosuppressive and immunomodulatory treatments have repeatedly improved outcomes in animal studies [13,26,27]. However, attempts to translate pharmacological immunosuppressive treatments from animal studies to human stroke patients have failed in the past, partly due to systemic side effects like opportunistic infections [28]. With respect to systemic side effects of pharmacological immunosuppressive stroke treatments, non-pharmacological approaches to modulate the postischemic immune response are particularly promising treatment strategies. Our current study suggests the use of low-salt diet and low-salt fluids for stroke patients as a feasible and well-tolerated approach to attenuate the postischemic inflammation of stroke patients.

In summary, our study firstly characterizes the migrasome as a novel pathomechanism and potential therapeutic target in acute ischemic stroke. The evidence of migrasomes in postmortem brain tissue of stroke patients highlights the clinical relevance of this finding. Above that, our study identifies dietary salt as a potential target to modulate outcomes of stroke patients.

## Materials and methods

### Animals

All experiments were performed in accordance with animal welfare regulations and experimental protocols were approved by the local governmental authorities (Landesamt für Natur, Umwelt und Verbraucherschutz, NRW, Germany). Adult male C57BL/6 mice at the age of 11 weeks were obtained from Charles River and housed in a controlled environment with a 12:12 h light-dark cycle.

### Experimental design

Mice received either high-salt diet containing 4% NaCl (ssniff, Germany) and tap water containing 1% NaCl ad libitum (sodium chloride-rich diet) or standard diet and tap water ad libitum (control), as previously described [3,4]. The body weight of all animals was monitored to verify that both groups consumed similar amounts of food and water. To exclude sodium-induced hypertension as a confounding factor, we performed regular blood pressure measurements. To assess the effect of sodium chloride-rich diet on blood sodium levels, we performed blood gas analyses.

### Middle cerebral artery occlusion

After seven days of high-salt diet, middle cerebral artery occlusion (MCAO) was induced under inhalation anesthesia with 1.5% isoflurane in 30% O_2_/70% N_2_O and maintenance of a constant body temperature of 37 °C ± 0.5 °C, as previously described [15]. In brief, the left common carotid artery and carotid bifurcation were exposed and the proximal left common and external carotid arteries were ligated. Retrograde perfusion of the left common carotid artery was transiently interrupted by a microvascular clip (FE691; Aesculap, Tuttlingen, Germany), the common carotid artery was incised and a silicon-coated 8–0 nylon monofilament (701956PK5Re, Doccol Corporation, Sharon, MA) was advanced into the middle cerebral artery. Middle cerebral artery occlusion was verified by laser Doppler (Periflux 5001; Perimed, Stockholm). Following 60 minutes of MCAO, the monofilament was retracted to allow reperfusion of the middle cerebral artery. Sham animals underwent the same treatment as animals in the high-salt diet group, except that the nylon filament was withdrawn immediately after insertion.

### Neurological deficit score assessment

We employed a modification of Menzies neuroscore, which ranges from 0 (no deficit) to 5 (death) for neurological deficit score assessment, as previously described [15]. Neurological deficit score assessment was performed by a blinded investigator.

### Tissue preparation

Three days after MCAO, mice were perfused through the left ventricle with phosphate buffered saline for 5 minutes and 4% paraformaldehyde for 10 minutes under deep xylazine/ketamine anesthesia. For immunohistochemistry, brains were removed, fixed in 4% paraformaldehyde over night, immersed in 20% sucrose for three days, frozen and stored at -80°C. Mice used for protein analyses were perfused with phosphate buffered saline and brains were processed immediately after perfusion.

### Infarct volume assessment

Coronal cryosections (10 μm) were collected at 300 μm intervals and stained with toluidine blue (Sigma, St Louis, MO). A blinded investigator measured the infarct area and the areas of ipsilateral hemisphere and contralateral hemisphere on each section using ImageJ software (public domain). An edema corrected infarct area was calculated as follows: Infarct area x (area of contralateral hemisphere / area of ipsilateral hemisphere). To calculate infarct volumes, the mean edema corrected infarct areas were multiplied by longitudinal diameters.

### Immunohistochemistry

Mounted coronal cryosections were firstly rinsed in 3% H_2_O_2_/Methanol for 10 minutes to block endogenous peroxidases and thereafter incubated in Blocking Reagent (Roche Diagnostics) for 15 minutes to prevent unspecific protein binding. Subsequently, we used the following primary antibodies: anti-NeuN (1:150, Millipore), anti-GFAP (1:500, Dako), anti-7/4 (1:200, Serotec), anti-Arginase-1 (1:500, Abcam), anti-F4/80 (1:500, Serotec), anti-TSPAN4 (1:100, LSBio), anti-O4 (1:50, Merck Millipore), and FluoroMyelin Green (1:300, ThermoFisher). NeuN was directly visualized with anti-mouse-488 (1:150, 45min, Life) and O4 was directly visualized with anti-mouse-AlexaFluor594 (1:100, 45min, Molecular Probes). To amplify the signal of GFAP, F4/80, 7/4, and TSPAN4, we applied HRP-conjugated streptavidin (DAKO, Denmark, 1:100, 45 min) and biotinyl tyramide (1:100, 15 min), after incubation with respective biotinylated secondary antibodies (biotinylated anti-rat antibody for F4/80 and 7/4 (1:100), biotinylated anti-mouse antibody for GFAP (1:100) biotinylated anti-rabbit antibody for Arginase-1 (1:100). Afterwards, amplified antigens were visualized with streptavidin-conjugated dye (Alexa Fluor594, Molecular Probes, 1:100, 45 min). Apoptotic cells were stained by terminal deoxynucleotidyl transferase dUTP nick-end labeling (TUNEL, Roche, Basel Ch). RNA-staining was performed using the SYTO RNASelectTM Green Fluorescent Cell Stain (S32703)-kit according to the manufacturers’ instructions. For nuclear counterstaining, we applied a mounting medium with 4´,6-diamidino-2-phenylindole (Vector, Burlingame, CA). Images were taken with a fluorescence microscope (Nikon Ecliplse 80i, Nikon GmbH, Düsseldorf, Germany) and ImageJ software (public domain) was used for cell counting.

### Electronmicroscopy

For fixation mice were transcardially perfused with 2% (v/v) formaldehyde and 2.5% (v/v) glutaraldehyde in 100 mM cacodylate buffer, pH 7.4, for morphological electron microscopic analysis or 2% (v/v) formaldehyde and 0.25% (v/v) glutaraldehyde in 100 mM cacodylate buffer, pH 7.4, for immunogold electron microscopic analysis. Tissue samples used for morphological analysis were postfixed in 0.5% (v/v) osmiumtetroxide and 1% (w/v) potassium hexacyanoferrate (III) in 0.1 M cacodylate buffer for 2 h at 4 °C followed by washing with distilled water. After dehydration (ascending ethanol 30 to 100%), specimens were incubated in propylenoxide (2x 15 min) and embedded in Epon using beem capsules. Tissue samples used for immunogold electron microscopic analysis were dehydrated in ethanol up to 70% and embedded in LR White medium (London Resin Company, UK). Ultrathin sections were collected on copper grids and negatively stained with 2% uranyl acetate for 10 min. For immunolabeling, grids were incubated on drops of primary antibodies (F4/80; NeuN; O4; actin, for details see IHC section) diluted 1:25 in PBS containing 2% (v/v) BSA-c (Aurion, The Netherlands) and 0.025% (v/v) Tween 20 (1 h at RT). After washing with the same solution, ultrathin sections were incubated with secondary antibodies conjugated to gold particles. Electron micrographs were taken at 60 kV with a Phillips EM-410 electron microscope using imaging plates (Ditabis, Pforzheim, Germany).

### Proteomic analyses

Brain tissue was homogenized mechanically as described previously [29] and then separated using a modified gradient and protocol according to a recently published study [30] with speeds of 14,000 g for 70 minutes. Particles were then stained with anti-F4/80-APC antibodies and after adding 10 μl of FlowCount fluorospheres / beads (Beckman Coulter; corresponding to 10,000 beads) the samples were sorted on a FACS-Aria III sorter (BD Biosciences) by first gating on particles smaller than 2 μm in size compared to beads and then on F4/80^+^-particles. Quantification was performed by comparing the number of beads with the number of F4/80^+^-particles.

For protein analysis, vesicles were lysed in 7 M urea, 2 M thiourea, 4% CHAPS, 50 mM DTT containing protease inhibitor (1 tablet Complete Mini EDTA free). Proteins were precipitated (2% DOC, TCA) for clean-up and re-dissolved in lysis buffer. Proteins were tryptically digested, peptides were extracted and subjected to mass spectrometric (MS) analysis. High-definition MS was performed using Synapt G2 SI ion mobility mass spectrometer coupled to M-Class UPLC (Waters Corp.) as described [31], with a 90 min gradient (solvent system 100% water versus 100% acetonitrile, both containing 0.1% formic acid; trap column V/M Symmetry C18 100 A 5 μm, 180 μm x 20 mm; reversed phase column HSS T3 1.8. μm 75 mm x 200 mm; 0.5 μl injection volume). Data were analyzed with Progenesis QI for proteomics software (Nonlinear Dynamics) and ProteinLynx Global Server (Waters). For subsequent considerations, the output was restricted to proteins with minimum three peptide hits and non-blood-related proteins. Proteins were grouped using Pantherdb.org.

### Microglial cell culture

The murine embryonic stem cell-derived microglial precursor cells (ESdMs) were placed at the disposal by Harald Neumann (University of Bonn) and culturing was performed as previously described [32,33]. Cells were treated only with LPS (500 ng/ml) or in combination with different NaCl concentrations (20 mM and 40 mM). Control groups were treated with the equivalent volume of solvent, i.e. DMSO.

### Primary murine microglia and astrocyte co-culture and time-lapse microscopy

Pups (1–5 days postpartum) from C57BL/6 mice were decapitated and after removal of the cerebellum the meninges were removed. Up to 5 brains were homogenized together in 5 ml L-glutamine-containing DMEM supplemented with 10% heat inactivated FCS, 1% non-essential amino acids, 1% penicillin/streptomycin and 0.1% β-mercaptoethanol with a 5 ml pipette. The homogenates were incubated for 5 minutes on ice. The resulting supernatants were pooled and centrifuged at 486 x g for 5 minutes at room temperature and the pellets were resuspended in medium. Cell suspension of two brains was transferred to a poly-L-lysin precoated 6-well plate and incubated at 37 °C and 5% CO_2_. After 1 and 7 days of culturing the medium was changed. Time-lapse microscopy was performed using a Zeiss AxioVert microscope. Subsequent video editing was done using Magix Video X3.

### Metabolic assays

For 72 h, ESdMs were incubated with LPS ± NaCl (40mM) to stimulate cells. Oxygen consumption rate (OCR) was determined under basal conditions and in response to 2 μM oligomycin, 1.5 μM FCCP, 100 nM rotenone plus 1 μM antimycin A (all Sigma-Aldrich). During OCR measurement activated ESdMs were cultured in XF-medium (XF Base Medium Minimal DMEM (Seahorse)) containing 10 mM glucose, 2 mM L-glutamin, and 1 mM sodium pyruvat (all from Sigma-Adlrich). OCR were evaluated using the Seahorse XFp Extracellular Flux Analyzer (Agilent Technologies) and analysed with Wave Software (Seahorse Bioscience).

### Enzyme-linked immunosorbent assay (ELISA)

ESdMs were incubated for either 72 h or 96 h with LPS ± NaCl (20 mM and 40 mM) to generate ESdMs supernatant. TNF-α and IL-6 cytokine detection was quantified using ELISA Ready-SET-Go (murine TNF-α/IL-6; eBioscience) according to the manufacturer’s instructions.

### Statistics

We used GraphPad Prism version 5 (GraphPad Software, La Jolla, CA) for statistical analyses. Functional outcomes were compared by 2way ANOVA, mortality rates were compared by Log-rank (Mantel-Cox) test and histological outcomes were compared by two-tailed unpaired t-test. All data are presented as mean ± s.e.m..

## Supporting information

S1 Video Time lapse video (contrast enhanced) of a microglia and astrocyte co-culture. After 30 min under normal conditions, salt (20 mM) is added to the medium. Note the fried egg-shaped microglia cell (marked green) within the box. After addition of salt, the astrocytes (red marked) start to shrink, maybe due to osmotic stress. However, multiple vesicle-like structures pop up and head in direction of the microglia cell over the time course of 3 hours.
(MP4)
